# Artificial intelligence annotated clinical-pathologic risk model to predict outcomes of advanced gastric cancer

**DOI:** 10.3389/fonc.2023.1099360

**Published:** 2023-03-28

**Authors:** Yan Chen, Lin Shou, Ying Xia, Yanju Deng, Qianguo Li, Zhishuang Huang, Youlan Li, Yanmei Li, Wenliang Cai, Yueshan Wang, Yingying Cheng, Hongzhuan Chen, Li Wan

**Affiliations:** Shatou Community Health Service Center, Shenzhen Hospital of Integrated Traditional Chinese and Western Medicine, Shenzhen, China

**Keywords:** artificial intelligence, SHAP (SHapley Additive exPlanations), advanced gastric cancer, risk model, treatment strategies

## Abstract

**Background:**

Gastric cancer with synchronous distant metastases indicates a dismal prognosis. The success in survival improvement mainly relies on our ability to predict the potential benefit of a therapy. Our objective is to develop an artificial intelligence annotated clinical-pathologic risk model to predict its outcomes.

**Methods:**

In participants (n=47553) with gastric cancer of the surveillance, epidemiology, and end results program, we selected patients with distant metastases at first diagnosis, complete clinical-pathologic data and follow-up information. Patients were randomly divided into the training and test cohort at 7:3 ratio. 93 patients with advanced gastric cancer from six other cancer centers were collected as the external validation cohort. Multivariable analysis was used to identify the prognosis-related clinical-pathologic features. Then a survival prediction model was established and validated. Importantly, we provided explanations to the prediction with artificial intelligence SHAP (Shapley additive explanations) method. We also provide novel insights into treatment options.

**Results:**

Data from a total 2549 patients were included in model development and internal test (median age, 61 years [range, 53-69 years]; 1725 [67.7%] male). Data from an additional 93 patients were collected as the external validation cohort (median age, 59 years [range, 48-66 years]; 51 [54.8%] male). The clinical-pathologic model achieved a consistently high accuracy for predicting prognosis in the training (C-index: 0.705 [range, 0.690-0.720]), test (C-index: 0.737 [range, 0.717-0.757]), and external validation (C-index: 0.694 [range, 0.562-0.826]) cohorts. Shapley values indicated that undergoing surgery, chemotherapy, young, absence of lung metastases and well differentiated were the top 5 contributors to the high likelihood of survival. A combination of surgery and chemotherapy had the greatest benefit. However, aggressive treatment did not equate to a survival benefit. SHAP dependence plots demonstrated insightful nonlinear interactive associations among predictors in survival benefit prediction. For example, patients who were elderly, or poor differentiated, or presence of lung or bone metastases had a worse prognosis if they undergo surgery or chemotherapy, while patients with metastases to liver alone seemed to gain benefit from surgery and chemotherapy.

**Conclusion:**

In this large multicenter cohort study, we developed an artificial intelligence annotated clinical-pathologic risk model to predict outcomes of advanced gastric cancer. It could be used to discuss treatment options.

## Introduction

A substantial number of patients with gastric cancer (GC) have synchronous distant metastases at the time of cancer diagnosis (1). Data from the surveillance, epidemiology, and end results (SEER) program suggests that about 30% of GC patients have distant metastases at first diagnosis (2, 3). The most common metastatic organs of advanced GC are peritoneum, liver, lung, and bone (2–4). The median survival time of patients with advanced GC is 3-7 months (5, 6). Despite great advances in its diagnosis and treatment over the past decades, the prognosis of advanced GC remains disappointing. Knowledge of the features regarding prognosis and therapeutic response is crucial to clinical decision-making and survival improvement. However, the clinical-pathologic characteristics associated with clinical outcomes have not been well defined in GC patients with distant metastases. Furthermore, an artificial intelligence annotated models for prognosis prediction of newly diagnosed advanced GC have not been reported. Hence, it would be important to investigate clinical-pathologic features regarding prognosis and therapeutic response, and develop an artificial intelligence model for prediction of clinical outcomes in advanced GC.

Nowadays, the optimal strategy for GC with advanced disease is still unknown. Chemotherapy is recommended as a cornerstone of treatment for advanced GC in the national comprehensive cancer network (NCCN) guideline and the fifth Japanese gastric cancer treatment guideline (7, 8). However, only a few advanced GC patients gain survival benefit from chemotherapy at the risk of adverse drug effects (9–13). Besides, there has been a long-running debate over whether surgical treatment is appropriate enough for patients with advanced GC (14–17). Some researchers suggested that surgical excision could improve overall survival (OS) for part of highly selected patients, while others hold different sounds. However, the common weakness of these studies was the small number of cases included. Therefore, a population-based study may be required to identify which patients could benefit from chemotherapy and surgery.

The success in survival improvement mainly depends on our ability to predict the potential benefit of a therapy (18, 19). Whether patients receive intensive treatments after cancer diagnosis often depends on the judgement of clinicians. However, such judgments often require extensive experience and are accompanied by randomness. Thus, risk assessment from objective evidence is particularly important for clinical decision-making. The artificial intelligence annotated model is widely accepted to provide assistance for clinical decision-making (20, 21). And there remains an opportunity to augment decision-making through artificial intelligence annotated tools for patients with advanced GC.

The shapley additive explanations (SHAP) is an artificial intelligence strategy based on game theory, which provides a unified method to interpreting machine learning models (20–22). It can be used to unlock the intrinsic importance of features for the prediction, such as, treatment decision-making.

The present study aimed to develop an artificial intelligence annotated clinical-pathologic risk model to predict clinical outcomes in a large multicenter cohort of 2642 GC patients with synchronous distant metastases. The prognosis-related clinical-pathologic features were identified from multivariable analysis. Next, a prognostic risk model with high performance was established and validated. More important, we provided explanations to the prediction with artificial intelligence SHAP method. We also provide novel insights into treatment options.

## Methods

### Patients and clinical characteristics

Data was obtained from SEER database using the SEERstat software version 8.3.5. The SEER database was the largest publicly available cancer dataset and collected cancer data from 21 population-based cancer registries covering about 34.6% of the United States population (23). This database included information about clinical-pathologic features and demographic information. The SEER database began registering information on the identification of organs with metastases in 2010. Importantly, cases from 2010 to 2016 had an adequate follow-up span, and the clinical-pathologic records were relatively complete. Thus, the present study enrolled GC patients diagnosed between 2010 and 2016.

Using these data, we identified 47553 patients with GC from 2010 to 2016. Given the inclusion and exclusion criteria, 2549 GC patients with synchronous distant metastases were identified. The exclusion criteria were as follows: age<18y or age>80y at the time of diagnosis; with more than one primary cancer; without pathological diagnosis; missing data of tumor location; missing data of metastatic organs to liver, lung, bone or brain; missing data of surgery; missing data of marital status; pathological type confirmed to be NET (Neuroendocrine tumor) stomach, sarcoma, GIST (Gastrointestinal stromal tumor) or lymphoma; missing data of differentiation status; missing data of TNM staging; M0 staging; without active follow-up.

**Figure 1:**
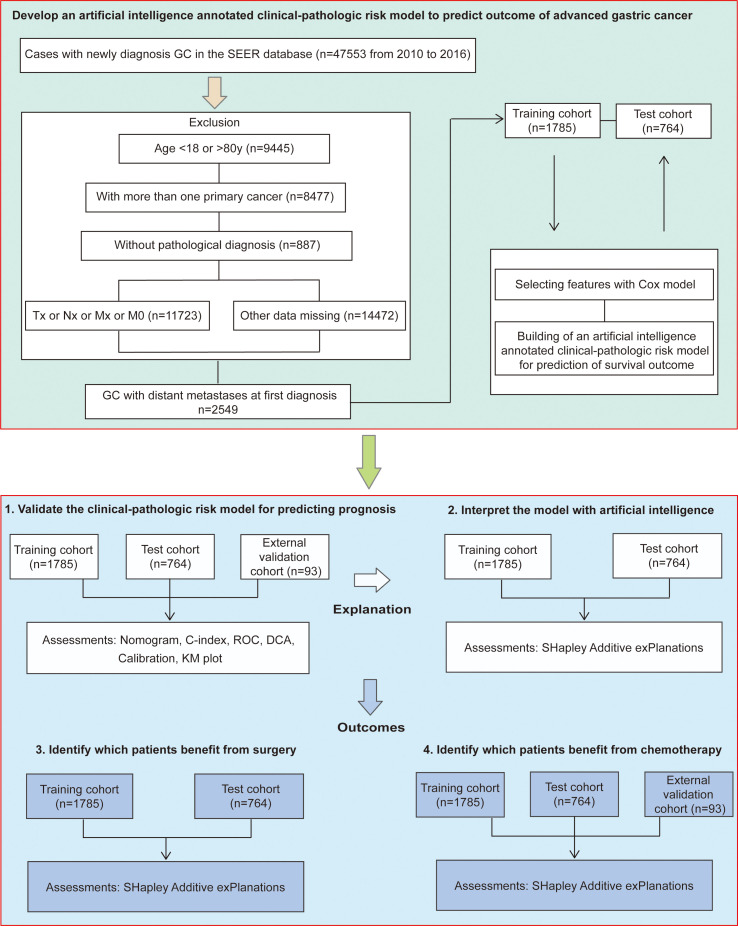
Study design.

Clinical-pathologic variables and demographic information were obtained for each case included age at diagnosis, sex, tumor location, differentiation status, tumor histology, TNM staging, site-specific metastasis to liver, lung or bone, surgery, chemotherapy, marital status, and follow-up data. Surgery included gastrectomy (surgery codes C10-C50) and gastrectomy plus metastectomy (surgery codes C60-C63). In addition, we collected another GC queue from The Cancer Genome Atlas (TCGA) and Gene Expression Omnibus (GEO) databases as the external validation cohort, which included 93 patients with distant metastasis at first diagnosis from six cancer centers (ACRG, YUHS, KUGH, KUCM, CGH, and TCGA-STAD); however, which organ with tumor metastasis or surgery information had not been reported in this cohort. Thus, it was only used to verify the predictive value of the available variables and identify patients who would benefit from chemotherapy. Data acquisition was described in our published studies (18, 19). The follow-up duration was measured from the time of diagnosis to the last follow-up date, and patients survived to the latest follow-up identified as censoring. The detailed study design was illustrated in **Figure 1**.

### Development and validation of the clinical-pathologic risk model

Data from a total 2549 GC patients with distant metastases at first diagnosis were randomly divided into the training (n=1785) and test cohort (n=764) at 7:3 ratio. Cox regression model was performed to identify the prognosis-related clinicopathologic features in the training cohort. Then, a clinical-pathologic risk model for prognosis prediction was established and validated in the validation cohorts (24). The performance of the prediction model was evaluated by discrimination and calibration, both of which were validated with 1,000 bootstrap samples. Harrell’s concordance index (C-index) was used to assess the discrimination performance of the clinical-pathologic risk model for prognostic prediction (25). Time dependent receiver operating characteristic (ROC) curve analysis was used to evaluate the 1,3,5-year OS predictive power of the risk model, and quantified by the area under the curve (AUC) in the training cohort, test cohort, and external validation cohort (26). And the calibration plots were performed to show consistency between the predicted probability and actual probability. Furthermore, decision curve analysis (DCA) was used to assess the clinical usefulness of predictive models.

### Model interpreting with artificial intelligence SHAP

To interpret how each clinicopathological feature and the clinical-pathologic risk model influenced the prognostic prediction in advanced GC, we used Shapley values. The artificial intelligence SHAP method, provided a unified method to interpreting machine learning models. Based on the SHAP package in python (27), we were able to get the importance of each clinicopathologic characteristic and the prediction model with interpretations on how they participated in the prediction of OS.

### Explanation of treatment decisions

SHAP dependence plots could demonstrate insightful nonlinear interactive associations among predictors. Consider that the success in survival improvement mainly depended on our ability to predict the potential benefit of a therapy, we evaluated the power of each clinicopathologic marker for predicting the surgery and chemotherapy benefit by Shapley values.

### Statistical analysis

Continuous variables were expressed as mean ± standard deviation (X ± SD), and compared among groups using t-test or Mann-Whitney test. Enumeration data were expressed as percentages and compared among groups by Chi-square or Fisher exact test. Survival estimates were obtained according to the Kaplan-Meier method and compared using the log-rank test. Variables, that reached significance with P < 0.05 in univariable analysis, were entered into the multivariable analysis using the cox proportional hazards model with an entry stepwise approach to identify covariates associated with increased all-cause mortality, and then hazard ratio with 95% confidence intervals (CIs) of each variable was achieved. Nomograms and calibration plots were generated using the R package ‘‘rms”. AUC and DCA were performed using the ‘‘pROC” and ‘‘dca.R” packages in R. The artificial intelligence SHAP was conducted with SHAP package in python. All statistical analyses were performed using R software (version 3.5.3), SPSS statistical software (version 26.0) and Python software (version 3.6). A two-sided P < 0.05 was considered statistically significant.

## Results

A total of 2549 cases diagnosed with advanced GC from SEER database as the training and test cohorts were included in this study. Another queue with 93 GC patients diagnosed with advanced disease was collected from TCGA and GEO database as the external validation cohort. For patients from SEER database, the median (interquartile range) age was 61 (53-69) years. 1725 (67.7%) patients were men, and 824 (32.3%) patients were women. The vast majority of tumors were poorly differentiated (77.0%) and located in the cardia (48.0%). Liver, lung, and bone metastases had been reported in 1047 (41.2%), 360 (14.1%), and 267 (10.5%) cases, respectively. 543 (21.3%) patients underwent surgery, and 1797 (70.5%) patients were treated with chemotherapy. Toward the last follow up, there were 2297 deaths and 252 censoring. Among them, patients who survived for more than one year were 818. Patients who survived for more than two years were 280, and who survived for more than three years were 110. The 1, 2, and 3-year OS were 32.09%, 10.98%, and 4.32%, separately. Besides, the distribution of clinicopathologic features and demographic information was similar between the training and test groups (**Table 1**). For patients in the external validation cohort, the median (interquartile range) age was 59 (48-66) years. 51 (54.8%) patients were men, and 42 (45.2%) patients were women. The detail clinicopathologic characteristics were listed in the **Supplementary Table 1**.

**Table 1:** Clinicopathologic characteristics of gastric cancer patients with distant metastases at first diagnosis.

Variables	Training cohort, n=1785	Test cohort, n=764	*P* value
Median age (range)	61 (52-69)	61 (53-70)	0.164
Male (%)	1200 (67.2)	525 (68.7)	0.245
Tumor location (%)			0.794
Cardia	857 (48.0)	367 (48.0)	
Body	249 (13.9)	96 (12.6)	
Antrum	420 (23.5)	185 (24.2)	
Whole	259 (14.5)	116 (15.2)	
Differentiation status (%)			0.383
Well or moderate	420 (23.5)	167 (21.9)	
Poor or undifferentiated	1365 (76.5)	597 (78.1)	
Tumor histology (%)			0.541
Adenocarcinoma	1317 (73.8)	554 (72.5)	
Signet ring cell or others	468 (26.2)	210 (27.5)	
Depth of invasion (%)			0.167
T1	496 (27.8)	233 (30.5)	
T2	138 (7.7)	52 (6.8)	
T3	521 (29.2)	195 (25.5)	
T4	630 (35.3)	284 (37.2)	
Lymph node metastasis (%)			0.110
N0	595 (33.3)	278 (36.4)	
N1	773 (43.3)	305 (39.9)	
N2	184 (10.3)	94 (12.3)	
N3	233 (13.1)	87 (11.4)	
Liver metastases (%)	720 (40.3)	327 (42.8)	0.253
Lung metastases (%)	239 (13.4)	121 (15.8)	0.107
Bone metastases (%)	184 (10.3)	83 (10.9)	0.672
Surgery (%)	378 (21.2)	165 (21.6)	0.935
Chemotherapy (%)	1278 (71.6)	519 (67.9)	0.065
Married (%)	1124 (63.0)	485 (63.5)	0.823

**Figure 2:**
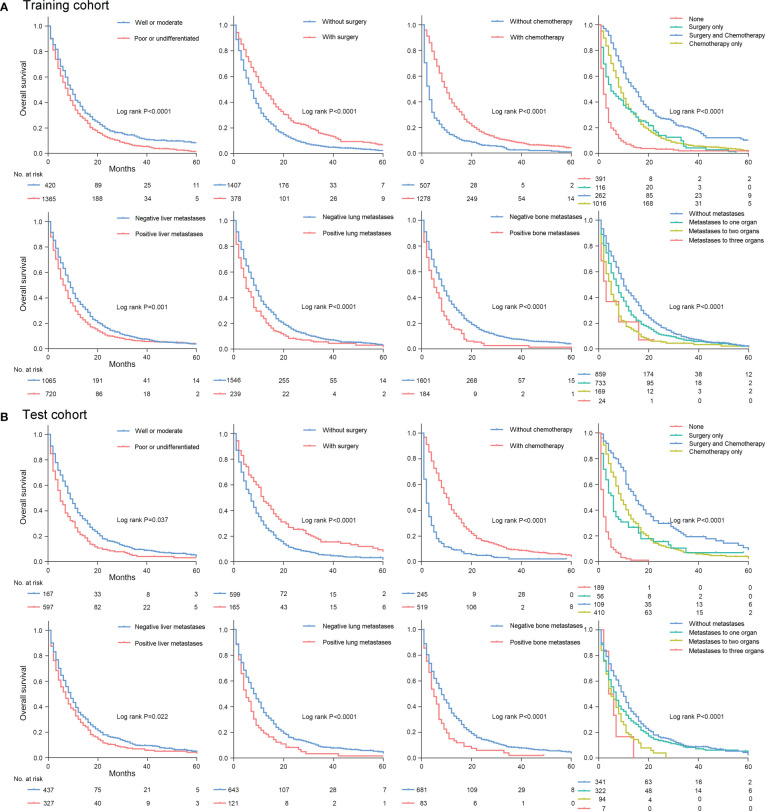
Kaplan-Meier plots for overall survival in the training cohort **(A)** and test cohort **(B)**.

Univariate analysis identified eight variables were significantly associated with all-cause mortality in the training and test cohorts, including age at diagnosis, differentiation status, lymph node metastasis, liver metastases, lung metastases, bone metastases, surgery, and chemotherapy (**Table S2**). After multivariable cox regression analysis, there remained seven clinicopathologic features (lymph node metastasis excluding), which were significantly associated with all-cause mortality in the training and test cohorts (**Table 2**). Among them, age≥65y, poor or undifferentiated, presence of liver metastases, presence of lung metastases, and presence of bone metastases were significantly associated with an increased all-cause mortality in the training and test cohorts (all P<0.05). In contrast, undergoing surgery and chemotherapy were significantly associated with a decreased all-cause mortality (all P<0.05). Kaplan-Meier plots demonstrated that these clinicopathologic features were the robust prognostic markers for OS in the training and test cohort (**Figures 2**, **S1**).

**Table 2:** Multivariable analysis for overall survival of gastric cancer patients with distant metastases at first diagnosis.

Variables	Training cohort, n=1785		Test cohort, n=764	
	Hazard Ratio (95% CI)	*P* Value	Hazard Ratio (95% CI)	*P* value
Age at diagnosis, years				
<65	1	Reference	1	Reference
≥65	1.131 (1.09-1.256)	0.021	1.238 (1.057-1.450)	0.008
Differentiation status				
Well or moderate	1	Reference	1	Reference
Poor or undifferentiated	1.426 (1.264-1.610)	<0.001	1.138 (1.036-1.249)	0.007
Lymph node metastasis				
N-	1	NA	1	NA
N+	0.954 (0.858-1.060)	0.376	0.940 (0.800-1.107)	0.459
Liver metastases				
No	1	Reference	1	Reference
Yes	1.171 (1.057-1.298)	0.003	1.083 (0.928-1.263)	0.311
Lung metastases				
No	1	Reference	1	Reference
Yes	1.295 (1.121-1.495)	<0.001	1.293 (1.051 -1.592)	0.015
Bone metastases				
No	1	Reference	1	Reference
Yes	1.449 (1.233-1.702)	<0.001	1.269 (1.003-1.613)	0.047
Surgery				
No	1	Reference	1	Reference
Yes	0.547 (0.480-0.623)	<0.001	0.441 (0.356-0.547)	<0.001
Chemotherapy				
No	1	Reference	1	Reference
Yes	0.321 (0.286-0.360)	<0.001	0.233 (0.194-0.280)	<0.001

Furthermore, considering that success in survival improvement mainly depends on therapy strategies, we further investigated the effect of different treatment combinations on the survival benefit of patients with advanced GC. For that purpose, treatment was reclassified into 4 categories according to surgery and chemotherapy: patients receiving both surgery and chemotherapy, or patients only receiving surgery, or patients only receiving chemotherapy, or patients without surgery and chemotherapy. In the entire cohort, 371 (14.6%) patients underwent both surgery and chemotherapy. 172 (6.7%) patients underwent surgery alone. 1426 (55.9%) patients underwent chemotherapy alone. And 580 (22.8%) did not receive either treatment. No significant difference was found in the number of therapeutic subclassification between the training and test cohort (**Table S3**). Kaplan-Meier plots revealed that patients with surgery plus chemotherapy treatment achieved the best prognosis, while patients without either treatment had the worst prognosis, and patients who received one of the treatments alone had an intermediate prognosis (**Figure 2**). And the median survival time in the training and test cohort was 15.0 months for patients treated with surgery plus chemotherapy, 9.0 months for patients treated chemotherapy alone, 4.0 months for patients treated with surgery alone, and 1.0 months for patients without treatments (**Table S4**). The median survival time in these groups showed significant statistical difference (all P<0.001).

Additionally, to investigate the effect of multiple organs metastases on survival, site-specific metastasis to liver, lung and bone was reclassified into 4 categories: patients with simultaneous metastases to three organs of liver, lung and bone, patients with simultaneous metastases to two organs of liver, lung or bone, patients with simultaneous metastases to one organ of liver, lung or bone, and patients without metastases to liver, lung or bone. In the entire cohort, 31 (1.2%) patients with simultaneous metastases to three organs, 263 (10.3%) patients with simultaneous metastases to any two organs, 1055 (41.4%) patients with metastases to one organ, and 1200 (47.1%) patients without metastases to liver, lung, or bone. No significant difference was found in the number of metastatic subclassification between the training and test cohort (**Table S3**). And the median survival time in the training and test cohort was 9.0 months for patients without metastases to liver, lung, or bone. The median survival time for patients with metastases to one organ was 7.0 months and 5.0 months in the training and test cohort. The median survival time in the training and test cohort was 4.0 months for patients with simultaneous metastases to any two organs. And the median survival time for patients with metastases to three organs was 2.0 months and 4.0 months in the training and test cohort (**Table S4**). The median survival time in these groups showed significant statistical difference (all P<0.001).

**Figure 3:**
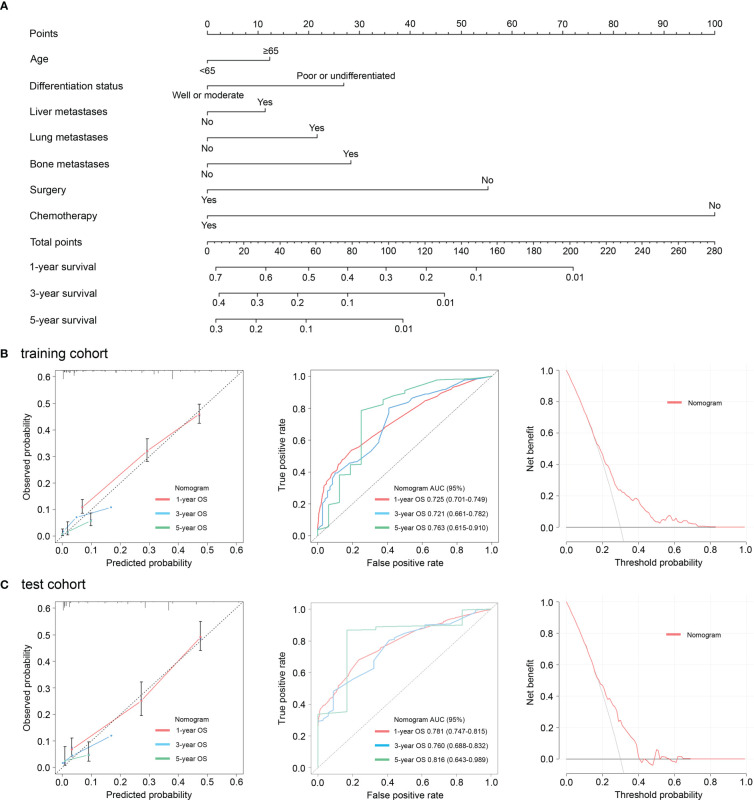
Nomograms incorporating the clinic-pathologic characteristics for evaluation of overall survival **(A)**. Calibration curves, receiver operating characteristic curves, and decision curves revealed good agreements between the predictive and actual probability in the training cohort **(B)**. Calibration curves, receiver operating characteristic curves, and decision curves revealed good agreements between the predictive and actual probability in the test cohort **(C)**.

Next, in order to predict the prognosis of patients with advanced GC more accurately and conveniently, we developed a clinical-pathologic risk model based on the independent prognostic features (including age at diagnosis, differentiation status, liver metastases, lung metastases, bone metastases, surgery, and chemotherapy) with significance in the multivariate analysis (**Figure 3A**). This clinical-pathologic model achieved a consistently high accuracy for predicting prognosis in the training (C-index: 0.705 [range, 0.690-0.720]) and test (C-index: 0.737 [range, 0.717-0.757]) cohorts. Moreover, good agreements between the predictive and actual probability were observed in calibration curves in the training cohort and validation cohort (**Figures 3B, C**). Furthermore, the AUC of the clinical-pathologic risk model for 1, 3 and 5-year OS evaluation was 0.725 (0.701–0.749), 0.721 (0.661–0.782), and 0.763 (0.615–0.910) in the training cohort (**Figure 3B**). Similar AUC values for the risk model were observed [0.781 (0.747–0.815) at 1-year OS, 0.760 (0.688–0.832) at 3-year OS and 0.816 (0.643–0.989) at 5-year OS] in the test cohort (**Figure 3C**). DCA plots also showed that the model had a good net benefit across the majority range of reasonable threshold probabilities in the training cohort and validation cohort (**Figures 3B, C**).

**Figure 4:**
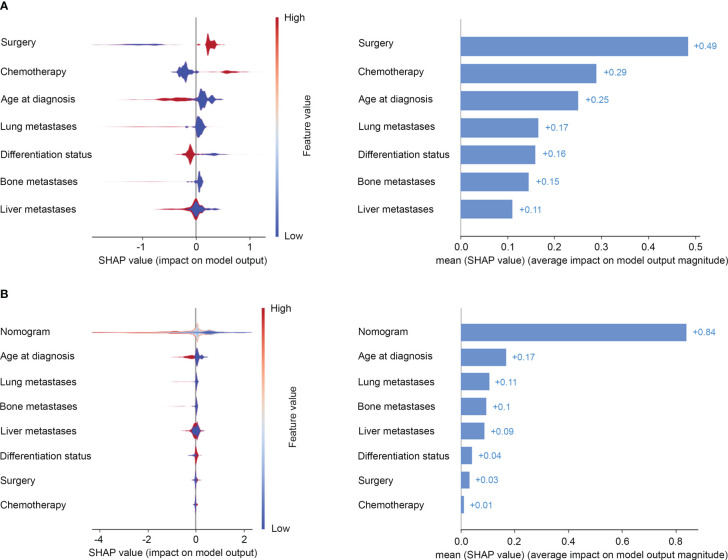
Risk estimates on clinical-pathologic features **(A)** and the risk model **(B)** by Shapley values to predict the risk of all-cause mortality. On the X-axis, the contribution of each feature is shown. A feature with a positive shapley value will favorably impact the prediction (decrease the risk of all-cause mortality). The influence of the value of the feature itself is shown on the Yaxis, for example, for nomogram, a high value (in red) is associated with a positive shapley value that will decrease the risk of all-cause mortality, while a low value (in blue) will increase the shapley value and the risk of all-cause mortality.

Risk estimates can be extracted from the prediction model by SHAP values then to allow explanation of risk on the global-level. The most important features to predict prognosis of advanced GC were surgery, chemotherapy, age at diagnosis, lung metastases, and differentiation status in SHAP interpretation (**Figure 4A**). Bone metastases and liver metastases were not among the top five features in prognosis prediction (**Figure 4A**). Furthermore, when seven clinical-pathologic features were integrated into a risk model, its importance to predict prognosis had been enhanced (**Figure 4B**).

**Figure 5:**
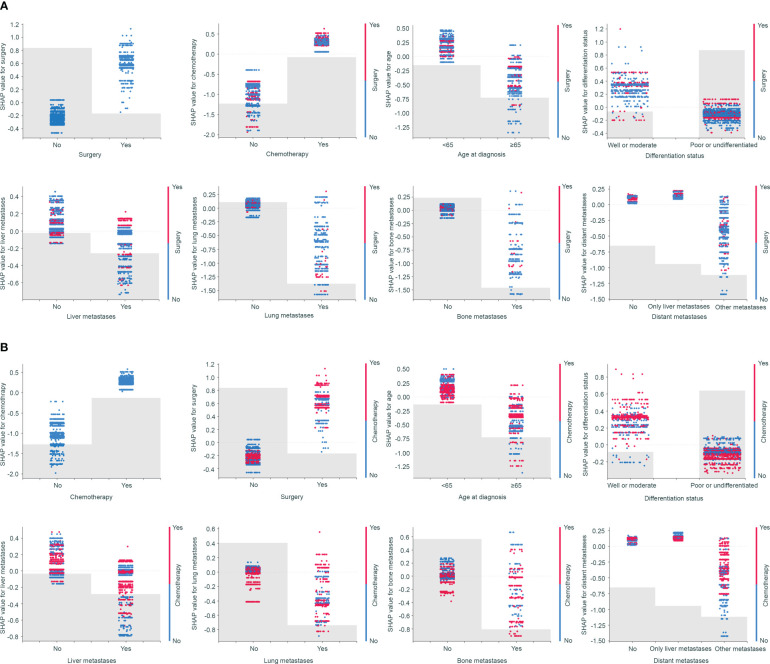
SHAP dependence plots demonstrated insightful nonlinear interactive associations among predictors in survival benefit prediction from surgery **(A)** and chemotherapy **(B)**.

SHAP dependence plots can demonstrate insightful nonlinear interactive associations among predictors in survival benefit prediction. In order to investigate the associations of different clinical-pathologic features with survival benefit from surgery or chemotherapy, we next performed SHAP interaction analyses. Results indicated that surgery and chemotherapy with a positive shapley value could improve overall survival (**Figure 5**). Especially, a combination of surgery and chemotherapy had the greatest benefit. However, aggressive treatment does not equate to a survival benefit. SHAP interaction plots revealed that patients who were elderly, or poor differentiated, or presence of lung or bone metastases had a worse prognosis if they undergo surgery or chemotherapy (**Figure 5**). Only patients with metastases to liver alone seemed to gain benefit from surgery or chemotherapy (**Figure 5**). This interesting finding and novel insights in treatment options could be used to discuss treatment options for advanced GC.

Additionally, we collected an external validation cohort from the TCGA and GEO databases. The results from the external validation cohort were consistent with our original reports from the training cohort and test cohorts. We found that age, differentiation status, and chemotherapy were also independent predictors for all-cause mortality (**Table S5** and **Figure S2**). Furthermore, a high accuracy (AUC: 0.744-0.940) in predicting 1, 3 and 5-year OS was observed in the external validation cohort (**Figure S3**). And the C-index was 0.694 (range, 0.562-0.826). SHAP interaction analyses indicated that chemotherapy could improve overall survival. However, patients who were elderly or with poor differentiated had a worse prognosis if they undergo chemotherapy in SHAP interpretation (**Figure S4**). The results were highly consistent in the training cohort, the test cohort, and the external validation cohort.

## Discussions

In this large multicenter cohort study, we investigated the clinicopathological features associated with prognosis in patients with advanced GC. We then developed and validated an artificial intelligence annotated clinical-pathologic risk model to predict outcomes of advanced GC. The clinical-pathologic model achieved a consistently high accuracy for predicting prognosis in the training and validation cohorts. Importantly, we provided explanations to the prediction with artificial intelligence SHAP method. We also provide novel insights into treatment options. We found that a combination of surgery and chemotherapy had the greatest benefit for advanced GC. However, aggressive treatment did not equate to a survival benefit. SHAP dependence plots demonstrated that patients who were elderly, or poor differentiated, or presence of lung or bone metastases had a worse prognosis if they undergo surgery or chemotherapy, while patients with metastases to liver alone seemed to gain benefit from surgery and chemotherapy. These findings could be used to discuss treatment options.

This study included 2549 patients with advanced GC from SEER database, whose median survival time was 7.0 months. The 1-year and 2-year survival rate was 32.09% and 10.98%. These results were almost consistent with Sarela’s report (5). The authors analyzed 55 consecutive GC patients with distant metastases, and found that the median survival time of these patients was 7 months. Similarly, the 1-year OS and 2-year OS were 35% and 16%, separately. However, a research including 116 advanced GC patients reported by Alici suggested that the median survival time was 3.9 months, and the 1-year OS was 19.8% (6). Compared with that in our study, patients in Alici’s study had a worse prognosis. It could be attributed to the reason as follow. All cases included in Alici’s study were poorly differentiated tumors, while 23% cases in our study were confirmed with well differentiated tumors.

The effect of age on the prognosis of GC remained controversial. Results from Tavares’s study suggested that compared with elder patients, the prognosis of young patients was significantly worse (28). However, this study only included 6 cases of young patients with advanced GC. Another small sample study with 55 patients indicated that age was not a prognostic factor for advanced GC (5). Conversely, an analysis from SEER database including 4596 signet-ring cell gastric carcinoma suggested that the prognosis of young GC patients was better (29). Our study also found that elderly patients with advanced GC had a worse prognosis. Similar to our study, poorly differentiated GC indicated a poor prognosis had been well reported (6, 30).

The most common metastatic organ for advanced GC was liver, follow by lung and bone (2–4, 31). There were 1047 patients presented with liver metastases, 360 with lung metastases and 267 with bone metastases in this study. A study published before indicated that nonperitoneal metastases were significantly associated with an increased all-cause mortality of stage IV GC (5). The same results also observed in the present study. We found that the presence of liver metastases, lung metastases, and bone metastases decreased the OS. In SHAP interpretation, lung metastasis was the most decisive factor leading to poor prognosis, then bone metastasis, followed by liver metastasis. This may be due to the important cardiopulmonary circulatory function of the lung, and the liver had a strong compensatory function, while the presence of bone metastases is often suggestive of other metastases simultaneously. Furthermore, patients with simultaneous multi-metastases to liver, lung and bone indicated worse prognosis.

The optimal strategy for GC with advanced disease was still unknown. Chemotherapy based on cisplatin and fluorouracil was recommended as the main treatment for stage IV GC in NCCN guideline and the fifth Japanese gastric cancer treatment guideline (7, 8). Chemotherapy may improve survival by half a year. However, only a few advanced GC patients gained survival benefit from chemotherapy at the risk of adverse drug effects (9–11). Furthermore, there has been a long-running debate over whether surgical treatment was appropriate enough for patients with advanced GC (14–16). With the development of conversion therapy, perioperative nutritional support and medical equipment, palliative operation and potential radical surgery may be gradually applicable to advanced GC. Increasing studies suggested that surgery may improve the OS of some patients with stage IV GC (14, 15). Our study based on a population-level found that chemotherapy and surgery can increase long time survival for patients with advanced GC, especially a combination of both. However, aggressive treatment did not equate to a survival benefit. SHAP dependence plots demonstrated that patients who were elderly, or poor differentiated, or presence of lung or bone metastases had a worse prognosis if they undergo surgery or chemotherapy, while patients with metastases to liver alone seemed to gain benefit from surgery and chemotherapy. These findings could be used to discuss treatment options.

Although several studies had proposed their nomograms for evaluation of prognosis in metastatic GC, they only focused on patients with liver metastases, or multi-organ metastases, or metachronous metastases, or palliative gastrectomy, which was different from our study (32–35). Furthermore, these studies only provided prognostic models, and lacked in-depth exploration of treatment options for advanced GC. Compared with the published researches, the present study had the following superiorities. Firstly, this artificial intelligence annotated model presented the best predictive performance, with the highest AUC value. Secondly, this was the largest multicenter study, including three cohorts with 2642 advanced GC. Importantly, we provided explanations to the prediction of prognosis and therapeutic response using artificial intelligence SHAP strategy. We also provided novel insights into treatment options, which could be used to discuss clinical decision-making.

However, the present study still has several limitations. Firstly, information on comorbidities and performance status was not available in the SEER database; Secondly, data on whether other organs with metastases had not been provided, like peritoneal metastases. Thirdly, more detail information about surgery and chemotherapy were not reported in the SEER database; Fourthly, this was a retrospective study need further verification. Although our study had some limitations, it was the first study to develop an artificial intelligence annotated clinical-pathologic risk model to predict outcomes of advanced GC and provide novel insights into treatment options.

In conclusion, this large multicenter cohort study identified the clinicopathological features associated with prognosis in patients with advanced GC, and established an artificial intelligence annotated clinical-pathologic risk model to predict outcomes of advanced GC with high performance. It also provided explanations to the prediction with artificial intelligence SHAP method and provided novel insights into treatment options. These findings could be used to discuss treatment options and may be used to optimize individual decision-making in patients with advanced GC.

## Data availability statement

The original contributions presented in the study are included in the article/**Supplementary Material**. Further inquiries can be directed to the corresponding authors.

## Ethics statement

The studies involving human participants were reviewed and approved by Shenzhen Hospital of Integrated Traditional Chinese and Western Medicine. Written informed consent for participation was not required for this study in accordance with the national legislation and the institutional requirements.

## Author contributions

All authors listed had made a substantial contribution to the work. LW, HC, and YC put forward the conception and designed the study. YC, YYC, YML and YX collected and collated the data. YC and LS analyzed data and wrote the manuscript together. YD, QL, ZH, WC, YW, and YLL made contribution to proofread the article. Finally, all the authors take responsible to the final manuscript and approved it for publication.
