# Supplementary material for: Artificial intelligence annotated clinical-pathologic risk model to predict outcomes of advanced gastric cancer
Source: Front Oncol. 2023 Mar 28;13:1099360. doi: 10.3389/fonc.2023.1099360 (PMC10086433; doi:10.3389/fonc.2023.1099360)
Supplement: Supplementary file 1 [file DataSheet_1.docx]

Supplementary Material

| **Table S1. Clinicopathologic characteristics of gastric cancer patients with distant metastases at first diagnosis.** | |
| --- | --- |
| **Variables** | **External validation cohort, n=93** |
| **Median age (range)** | 59 (48-66） |
| **Male (%)** | 51 (54.8) |
| **Tumor location (%)** |  |
| Cardia | 14 (15.1) |
| Body | 22 (23.7) |
| Antrum | 22 (23.7) |
| Whole | 9 (9.7) |
| NA | 26 (28.0) |
| **Differentiation status (%)** |  |
| Well or moderate | 16 (17.2) |
| Poor or undifferentiated | 56 (60.2) |
| NA | 21 (22.6) |
| **Tumor** **histology (%)** |  |
| Adenocarcinoma | 58 (62.4) |
| Signet ring cell or others | 35 (37.6) |
| **Depth of invasion (%)** |  |
| T1 | 0 |
| T2 | 18 (19.4) |
| T3 | 29 (31.2) |
| T4 | 27 (29.0) |
| NA | 19 (20.4) |
| **Lymph node metastasis (%)** |  |
| N0 | 7 (7.5) |
| N1 | 18 (19.4) |
| N2 | 27 (29.0) |
| N3 | 22 (23.7) |
| NA | 19 (20.4) |
| **Chemotherapy (%)** |  |
| Yes | 10 (10.8) |
| No | 36 (38.7) |
| NA | 47 (50.5) |

NA indicated miss value

| **Table S2. Univariate analysis for overall survival of gastric cancer patients with distant metastases at first diagnosis.** | | | | |
| --- | --- | --- | --- | --- |
| **Variables** | **Training cohort, n=1785** | | **Test cohort, n=764** | |
|  | **HR (95% CI)** | ***P* value** | **HR (95% CI)** | ***P* value** |
| **Age at diagnosis, Y** |  |  |  |  |
| <65 | 1 | Reference | 1 | Reference |
| ≥65 | 1.226 (1.107-1.357) | <0.001 | 1.462 (1.252-1.707) | <0.002 |
| **Sex** |  |  |  |  |
| Female | 1 | Reference | 1 | Reference |
| Male | 1.049 (0.945-1.164) | 0.372 | 1.109 (0.944-1.304) | 0.208 |
| **Tumor location** |  |  |  |  |
| Whole | 1 | Reference | 1 | Reference |
| Cardia | 0.936 (0.807-1.079) | 0.350 | 0.945 (0.760-1.174) | 0.610 |
| Body | 0.940 (0.783-1.129) | 0.509 | 0.909 (0.685-1.207) | 0.510 |
| Antrum | 0.882 (0.750-1.039) | 0.132 | 0.827 (0.645-1.059) | 0.133 |
| **Differentiation status** |  |  |  |  |
| Well or moderate | 1 | Reference | 1 | Reference |
| Poor or undifferentiated | 1.268 (1.128-1.425) | <0.001 | 1.102 (1.006-1.208) | 0.037 |
| **Tumor histology** |  |  |  |  |
| Adenocarcinoma | 1 | Reference | 1 | Reference |
| Signet ring cell or others | 1.139 (1.020-1.273) | 0.021 | 1.057 (0.895-1.249) | 0.512 |
| **Depth of invasion** |  |  |  |  |
| T1-2 | 1 | Reference | 1 | Reference |
| T3-4 | 0.905 (0.817-1.002) | 0.055 | 0.895 (0.767-1.045) | 0.162 |
| **Lymph node metastasis** |  |  |  |  |
| N- | 1 | Reference | 1 | Reference |
| N+ | 0.891 (0.804-0.988) | 0.029 | 0.779 (0.667-0.910) | 0.002 |
| **Liver metastases** |  |  |  |  |
| No | 1 | Reference | 1 | Reference |
| Yes | 1.182 (1.070-1.305) | 0.001 | 1.193 (1.026-1.388) | 0.022 |
| **Lung metastases** |  |  |  |  |
| No | 1 | Reference | 1 | Reference |
| Yes | 1.445 (1.255-1.664) | <0.001 | 1.506 (1.230-1.844) | <0.001 |
| **Bone metastases** |  |  |  |  |
| Yes | 1 | Reference | 1 | Reference |
| No | 1.450 (1.2395-1.697) | <0.001 | 1.572 (1.240-1.993) | <0.001 |
| **Surgery** |  |  |  |  |
| No | 1 | Reference | 1 | Reference |
| Yes | 0.613 (0.542-0.694) | <0.001 | 0.583 (0.483-0.705) | 0.000 |
| **Chemotherapy** |  |  |  |  |
| No | 1 | Reference | 1 | Reference |
| Yes | 0.361 (0.324-0.403) | <0.001 | 0.288 (0.243-0.340) | <0.001 |

| **Table S3. Subclassification for advanced gastric patients** | | | | | |
| --- | --- | --- | --- | --- | --- |
| **Variables** |  | **Training cohort, n=1785** |  | **Validation cohort, n=764** | ***P*** |
| **Treatment** |  |  |  |  | 0.319 |
| Without surgery and chemotherapy |  | 391 (21.9%) |  | 189 (24.7%) |  |
| Chemotherapy only |  | 1016 (56.9%) |  | 410 (53.7%) |  |
| Surgery only |  | 116 (6.5%) |  | 56 (7.3%) |  |
| Both surgery and chemotherapy |  | 262 (14.7%) |  | 109 (14.3%) |  |
| **Number of metastases to liver, lung and bone** |  |  |  |  | 0.121 |
| 0 |  | 859 (48.1%) |  | 341 (44.6%) |  |
| 1 |  | 733 (41.1%) |  | 322 (42.1%) |  |
| 2 |  | 169 (9.5%) |  | 94 (12.3%) |  |
| 3 |  | 24 (1.3%) |  | 7 (0.9%) |  |

| **Table S4. Median Survival of patients with advanced gastric cancer in subclassification** | | | | | | |
| --- | --- | --- | --- | --- | --- | --- |
| **Variables** |  | **Training cohort, n=1785** | |  | **Validation cohort, n=764** | |
|  |  | **Median, mo** | **P Value** |  | **Median (IQR), mo** | ***P* Value** |
| **Treatment** |  |  | <0.001 |  |  | <0.001 |
| Without surgery and chemotherapy |  | 1.0 (0.748-1.252) |  |  | 1.0 (0.762-1.238) |  |
| Chemotherapy only |  | 9.0 (8.419-9.581) |  |  | 9.0 (7.986-10.014) |  |
| Surgery only |  | 4.0 (2.518-5.482) |  |  | 4.0 (2.197-5.803) |  |
| Both surgery and chemotherapy |  | 15.0 (12.776-17.224) |  |  | 15.0 (11.809-18.191) |  |
| **Number of metastases to liver, lung and bone** |  |  | <0.001 |  |  | <0.001 |
| 0 |  | 9.0 (8.274-9.726) |  |  | 9.0 (7.713-10.287) |  |
| 1 |  | 7.0 (6.276-7.724) |  |  | 5.0 (3.827-6.173) |  |
| 2 |  | 4.0 (3.002-4.998) |  |  | 4.0 (2.654-5.346) |  |
| 3 |  | 2.0 (0.948-3.052) |  |  | 4.0 (1.434-6.566) |  |

| **Table S5. Univariate and multivariable analysis for overall survival in external validation cohort.** | | | | |
| --- | --- | --- | --- | --- |
| **Variables** | **Univariate analysis** | | **Multivariable analysis** | |
|  | **HR (95% CI)** | ***P* value** | **HR (95% CI)** | ***P* value** |
| **Age at diagnosis, Y** |  |  |  |  |
| <65 | 1 | Reference | 1 | Reference |
| ≥65 | 1.941 (1.220-3.089) | 0.005 | 1.042 (1.001-1.084) | 0.044 |
| **Sex** |  |  |  |  |
| Female | 1 | Reference | NA | NA |
| Male | 0.978 (0.616-1.552) | 0.925 | NA | NA |
| **Tumor location** |  |  |  |  |
| Cardia | 1 | Reference | 1 | Reference |
| Body | 0.731 (0.424-1.262) | 0.261 | NA | NA |
| Antrum | 0.904 (0.570-1.435) | 0.670 | NA | NA |
| Whole | 1.283 (0.825-1.995) | 0.269 | NA | NA |
| **Differentiation status** |  |  |  |  |
| Well or moderate | 1 | Reference | 1 | Reference |
| Poor or undifferentiated | 2.462 (1.201-5.049) | 0.014 | 3.345 (0.925-12.093) | 0.066 |
| **Tumor histology** |  |  |  |  |
| Adenocarcinoma | 1 | Reference | NA | NA |
| Signet ring cell or others | 1.418 (0.892-2.253) | 0.139 | NA | NA |
| **Depth of invasion** |  |  |  |  |
| T1-2 | 1 | Reference | NA | NA |
| T3-4 | 1.215 (0.686-2.154) | 0.504 | NA | NA |
| **Lymph node metastasis** |  |  |  |  |
| N- | 1 | Reference | NA | NA |
| N+ | 1.222 (0.488-3.060) | 0.668 | NA | NA |
| **Chemotherapy** |  |  |  |  |
| No | 1 | Reference | 1 | Reference |
| Yes | 0.443 (0.227-0.863) | 0.003 | 0.110 (0.013-0.944) | 0.044 |


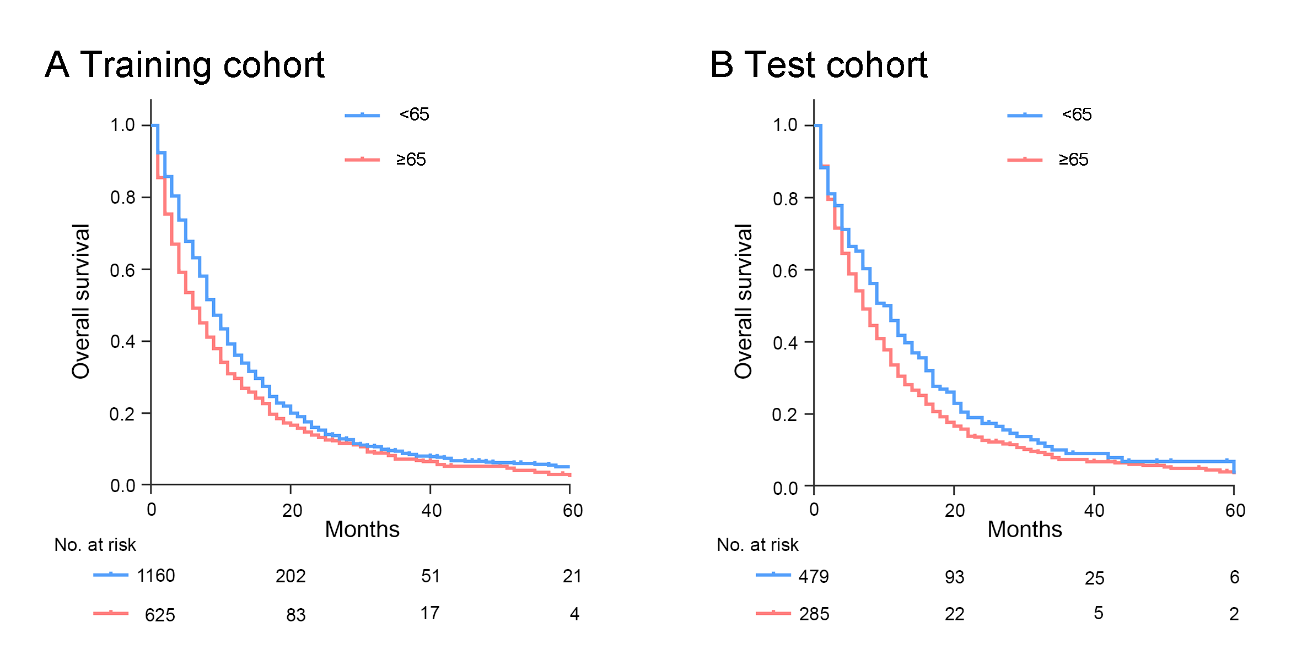


**Figure S1.** Kaplan–Meier plots for overall survival in the training cohort (A) and test cohort (B).


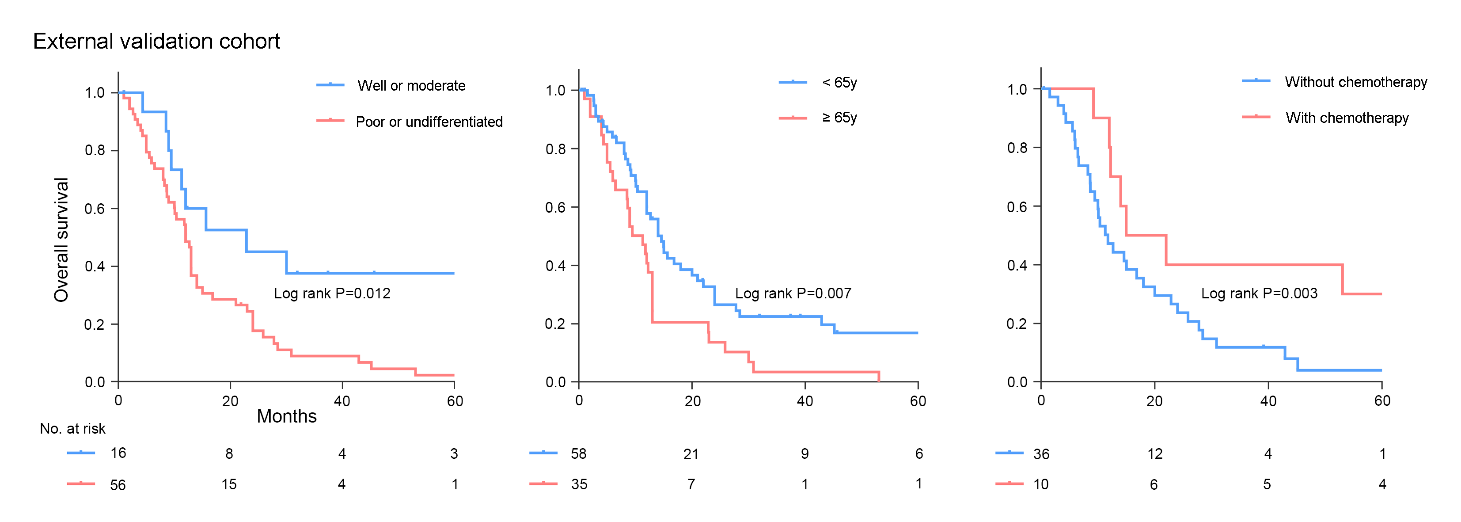


**Figure S2.** Kaplan–Meier plots for overall survival in the external validation cohort.


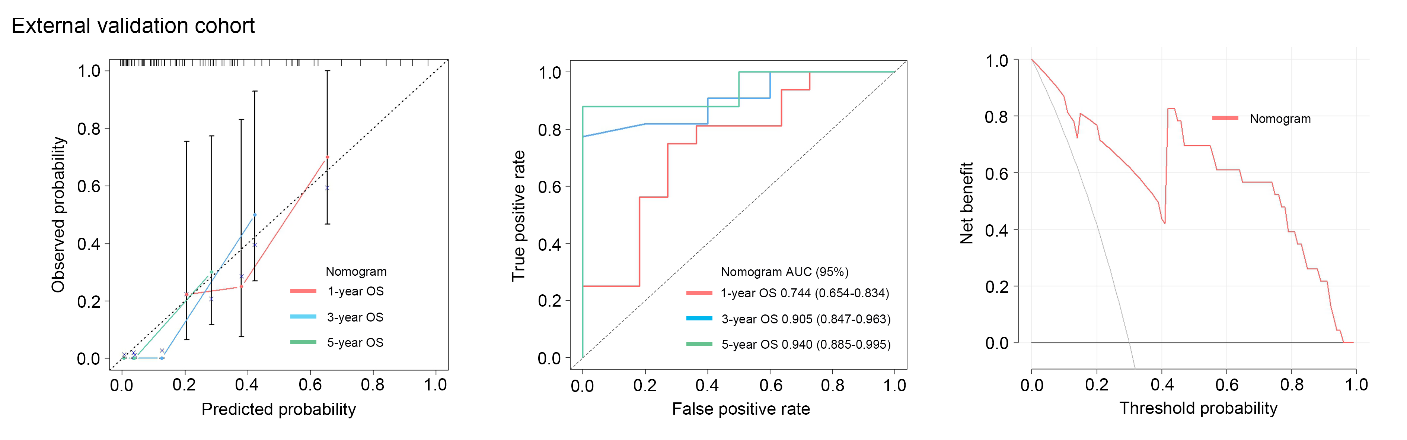


**Figure S3.** Calibration curves, receiver operating characteristic curves, and decision curves revealed good agreements between the predictive and actual probability in the external validation cohort.


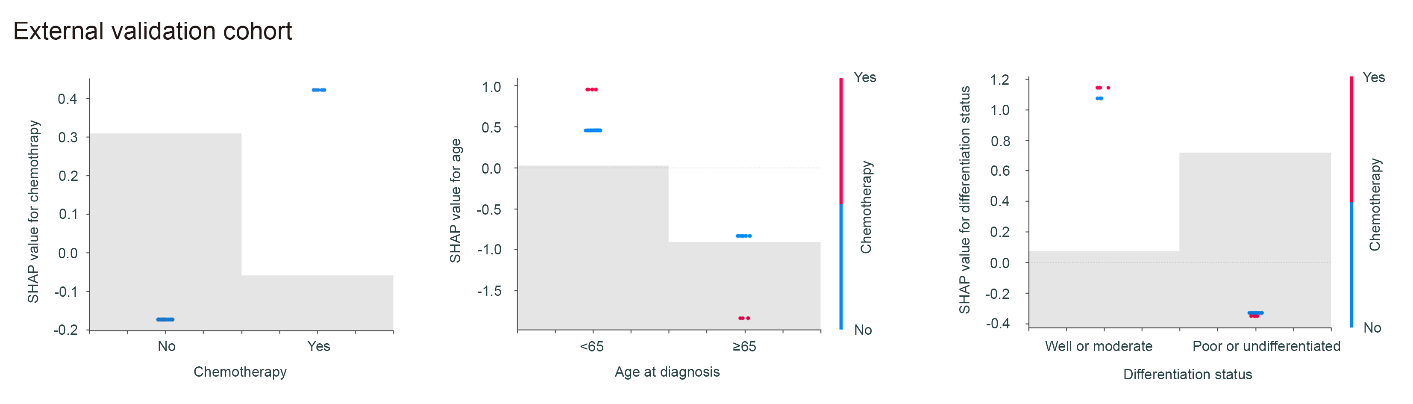


**Figure S4.** SHAP dependence plots demonstrated insightful nonlinear interactive associations among predictors in survival benefit prediction from chemotherapy in the external validation cohort.
